# Supplementary material for: Splice-Junction-Based Mapping of Alternative Isoforms in the Human Proteome
Source: Cell Rep. Author manuscript; Available in PMC 2020 Jan 15. (PMC6961840; doi:10.1016/j.celrep.2019.11.026)
Supplement: 2 [file NIHMS1546469-supplement-2.zip › DF1/PXD010154/Liver/Liver_53_SDC3_YPALAQGAAHGAGAGAGAAAGPGAR.pdf]

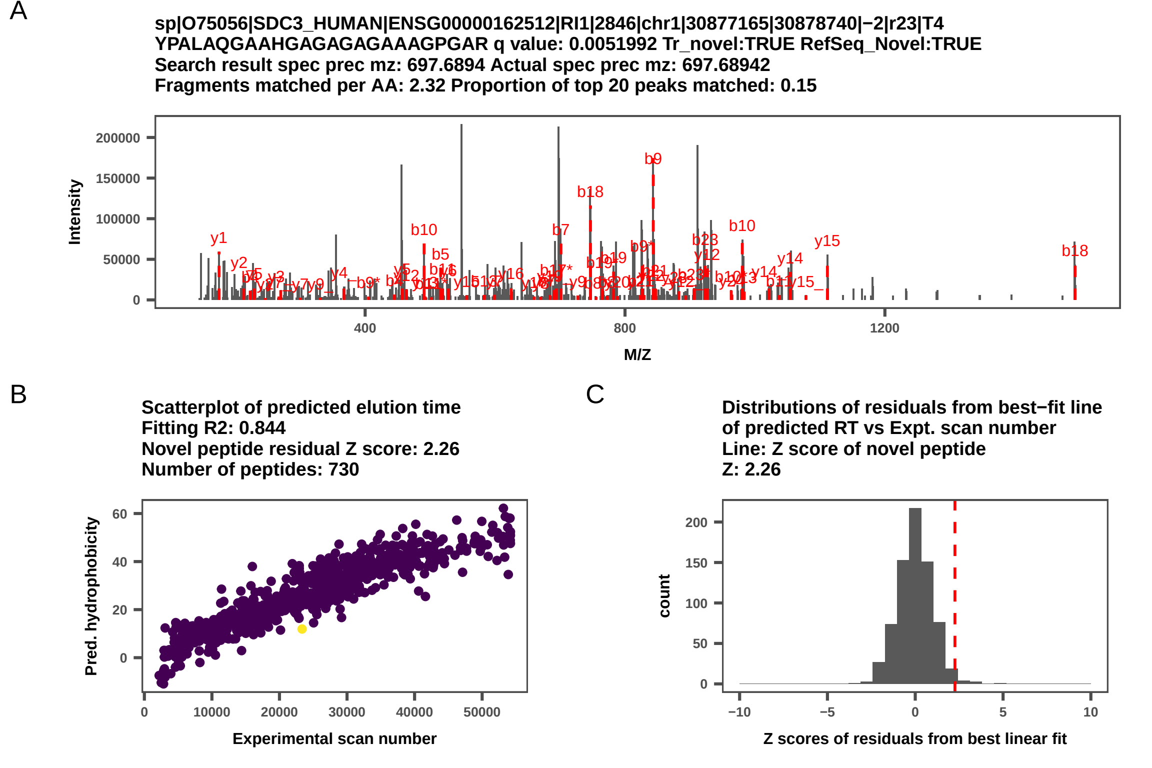
A
sp|O75056|SDC3_HUMAN|ENSG00000162512|RI1|2846|chr1|30877165|30878740|−2|r23|T4
YPALAQGAAHGAGAGAGAAAGPGAR q value: 0.0051992 Tr_novel:TRUE RefSeq_Novel:TRUE
Search result spec prec mz: 697.6894 Actual spec prec mz: 697.68942
Fragments matched per AA: 2.32 Proportion of top 20 peaks matched: 0.15
Intensity
200000
150000
100000
50000
0
400
800
1200
M/Z
b9
b18
b10
b7
b10
y1
y15
b18
b23
y12
b5
y14
y2
B
Scatterplot of predicted elution time
Fitting R2: 0.844
Novel peptide residual Z score: 2.26
Number of peptides: 730
Pred. hydrophobicity
60
40
20
0
0
10000
20000
30000
40000
50000
Experimental scan number
C
Distributions of residuals from best−fit line
of predicted RT vs Expt. scan number
Line: Z score of novel peptide
Z: 2.26
count
200
150
100
50
0
−10
−5
0
5
10
Z scores of residuals from best linear fit
